# Modelling the Proportion of Influenza Infections within Households during Pandemic and Non-Pandemic Years

**DOI:** 10.1371/journal.pone.0022089

**Published:** 2011-07-14

**Authors:** Kin On Kwok, Gabriel M. Leung, Steven Riley

**Affiliations:** 1 Department of Community Medicine and School of Public Health, The University of Hong Kong, Pokfulam, Hong Kong Special Administrative Region, China; 2 MRC Centre for Outbreak Analysis and Modelling, Department of Infectious Disease Epidemiology, School of Public Health, Imperial College London, London, United Kingdom; Center for Complex Networks and Systems Research - Indiana University at Bloomington, United States of America

## Abstract

**Background:**

The key epidemiological difference between pandemic and seasonal influenza is that the population is largely susceptible during a pandemic, whereas, during non-pandemic seasons a level of immunity exists. The population-level efficacy of household-based mitigation strategies depends on the proportion of infections that occur within households. In general, mitigation measures such as isolation and quarantine are more effective at the population level if the proportion of household transmission is low.

**Methods/Results:**

We calculated the proportion of infections within households during pandemic years compared with non-pandemic years using a deterministic model of household transmission in which all combinations of household size and individual infection states were enumerated explicitly. We found that the proportion of infections that occur within households was only partially influenced by the hazard *h* of infection within household relative to the hazard of infection outside the household, especially for small basic reproductive numbers. During pandemics, the number of within-household infections was lower than one might expect for a given 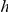 because many of the susceptible individuals were infected from the community and the number of susceptible individuals within household was thus depleted rapidly. In addition, we found that for the value of 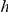 at which 30% of infections occur within households during non-pandemic years, a similar 31% of infections occur within households during pandemic years.

**Interpretation:**

We suggest that a trade off between the community force of infection and the number of susceptible individuals in a household explains an apparent invariance in the proportion of infections that occur in households in our model. During a pandemic, although there are more susceptible individuals in a household, the community force of infection is very high. However, during non-pandemic years, the force of infection is much lower but there are fewer susceptible individuals within the household.

## Introduction

The emergence [1] and global spread [2], [3] of a novel human strain of influenza A during 2009 highlighted the need for a strong evidence base to support health policy during the early stages of global infectious disease outbreaks. Even though the burden of illness was substantial [4], the 2009 strain was much less severe than previous pandemics [5], in particular compared to 1918 [6]. From a policy perspective, a less severe pandemic was challenging because costly interventions could not be justified. With hindsight, the 2009 pandemic served mainly to highlight the need for the scientific community and public health planners to rapidly and accurately assess the severity of an emerging respiratory disease. However, despite the mild recent pandemic, it seems likely from the initial public response that more expensive interventions such as household quarantine and antiviral prophylaxis would be implemented in many countries during any future moderate or severe pandemics.

The efficacy of household-based mitigation strategies depends on the proportion of infections that occur within households during a pandemic [7]. If this proportion is high, then the overall force of infection experienced by the community at large will be much reduced by effective household isolation or quarantine. If the proportion is low, then household-based mitigation strategies will be less effective. Unfortunately, estimating the proportion of infection that actually does occur within households is challenging.

Current estimates that approximately 30% of transmission events occur between members of the same household [8]–[9] are based on studies of non-pandemic influenza. In the absence of any empirical studies from 2009 or earlier pandemic, we conducted a theoretical study of factors that could contribute to substantial differences between the proportion of infections that take place within households during pandemic and non-pandemic years. For example, the distribution of susceptible individuals in households is different in the two scenarios. During a genuine pandemic, all individuals are susceptible and there is no clustering in households. However, during non-pandemic years, some individuals are immune due to prior infection or vaccination and their distribution by household is likely to be clustered: immune individuals are more likely to have been infected by a fellow household member during a previous year than another random member of the population. One would thus expect susceptible individuals to be clustered away from immune individuals. Also, the duration and absolute magnitude of the force of infection from the community, which is defined as the hazard of infection experienced by one susceptible individual, is different during a pandemic season than during a non-pandemic season. The community force of infection should last longer and be greater during a pandemic.

In the study described here, we used deterministic mathematical models of influenza transmission over multiple seasons to investigate the trade-off between changes in the clustering of susceptible individuals within households and changes in the absolute force of infection from the community. Specifically, we sought to test the hypothesis that there could be substantial differences between the proportion of infections that occur within a household during pandemic and non-pandemic years even if a) the underlying transmissibility of influenza is constant and b) the infectiousness of individuals within the household relative to their infectiousness outside the household is constant.

## Methods

First, we used a compartmental SEIRS model [10], [11], [12] to simulate how individuals could be infected with influenza over multiple decades (Model A). In our simulation study, pandemic influenza occurred in the first year and non-pandemic influenza was assumed to be is the first year of a regular annual cycle of infection. The main dynamic difference between the two types of influenza season was that the entire population was susceptible in pandemic whereas was immunity in a proportion of individuals in the population during non-pandemic years. We defined *N* as the total number of individuals in the population, 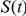 as the number who were susceptible to infection from the current strain at day 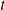, 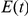 as the number who had been infected but were not yet infectious, 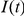 as the number who were infectious, and 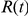 as the number who recovered and were presumed to be immune to the current strain. The force of infection is the hazard of infection experienced by a single susceptible individual. In our model we defined it to be 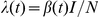, where 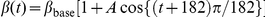 [12], 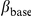 is a fundamental unit of transmission, and 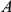 is the amplitude of seasonal forcing which is used to capture the seasonal oscillations in incidence by changing the effective transmission rate of the virus. This formulation implemented a season-based year of 52 seven-day weeks which is equivalent to 364 days per year with 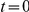 being the start of the first week at which the potential for influenza transmission is lowest: i.e., the middle of temperate summer.

Solutions were initiated at the midpoint of the summer before a pandemic winter. We assumed that when 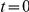, there was a single individual infectious with the pandemic strain (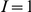) and all other individuals were susceptible (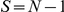). The rate of change of susceptible individuals was defined as 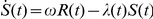, where 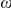 was the reciprocal of the average effective duration of immunity to influenza in days. Similarly, 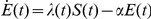, where 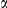 was the reciprocal of the average duration of the latent period in days; 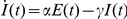, where 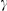 was the reciprocal of the duration of infection; and 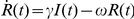. The solutions to the model equations were obtained numerically [13].

Non-household Model A was refined by household-based Model B using methods similar to those of [14]. We let 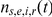 be the number of households at time 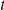 in which 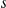 individuals were susceptible, 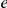 were exposed and incubating but not yet infectious, 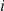 were infectious, and 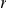 had recovered and were presumed to be immune. For example, the variable value 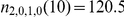 indicates that at day 10 there were an expected 120.5 households of size 3 in which 2 people were susceptible and 1 was infectious. We considered populations made up of households of sizes 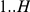, with the size of households of type 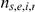 equal to 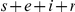. Using standard nomenclature for combinations, the total number of state variables required to describe all possible disease states for all household sizes was 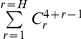 where 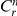 is the number of possible outcomes by drawing r objects from n objects.

To illustrate the model further, consider the special case of a population is composed entirely of households of size 1 or 2. There are 4 combinations for singleton households: n_1,0,0,0_ , n_0,1,0,0_ , n_0,0,1,0_ and n_0,0,0,1_ and 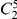 for households with size 2. Hence, for populations with household sizes up to 6, this model formulation required 209 variables of type 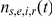. Note that the number of households at size 5 or above was collapsed as one number from Hong Kong census and statistics department. We assumed that the number of households at size 5 or above as the number of households at size 5. We defined the set of all possible household types (combinations of 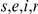) for a given 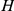 to be 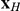, which allowed us to express the values of individual state variables 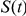, 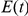, 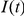, and 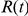 in terms of 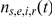, e.g.
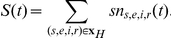
Therefore, the community force of infection is driven entirely by the sum of the household variables, i.e. there is no separate set of variables for the community dynamics,
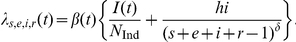
Parameter 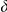 determined the degree to which household transmission was genuinely mass action compared with the degree to which it is pseudo-mass action. If transmission within households was genuinely mass action (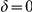), then members of very large households had the same chance of being infected by a single infectious individual as did members of very small households. If transmission was pseudo-mass action (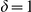), then the expected number of true secondary cases within a single fully susceptible household is the same for both large and small households: i.e. infectivity is shared evenly amongst those present. Parameter 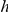 was the scaling ratio for the force of infection within households relative to that that between households and does have a direct intuitive interpretation if we consider a special case in which *δ* = 1 and, for one household in our population, the proportion of infectious individuals within the household is equal to the proportion of infectious in the community. Under these circumstances, if *h* were less than 1, then the force of infection from the community would be greater than the force of infection from the household. If *h* were greater than 1, the balance of infectious hazards would be reversed.

The dynamic model for a population of households was defined by a master equation for the time derivative of 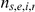,
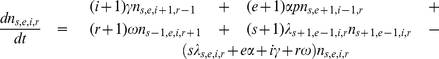
in which the first four terms corresponded to a different event (recovery, becoming infectious, loss of immunity and infection). We assumed that 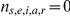 if the sum 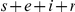 was less than 1 or greater than 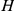 or if any 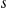, 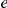, 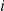 or 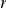 was less than 1 or greater than 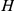. Note that Model A is a nested sub-model of Model B: if either the maximum household size is 1 (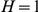) or there was no transmission within households (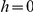), the two models were equivalent.

In general, the basic reproductive number for household models of infectious disease transmission is difficult to define precisely in a closed form [15], [16]. To ensure that our results were comparable with those from previous studies of influenza transmission (e.g. [7], [12], [17], [18], [19]), we constrained the cumulative attack rate to be 57.9% in the first year even in the presence of seasonal forcing. Based on the simple relationship between the attack rate (AR) and basic reproductive number (Ro) for a mass action system, 
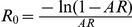
, this was equivalent to a basic reproductive number 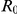 of 1.5 in Model A [20] .

**Figure 1:**
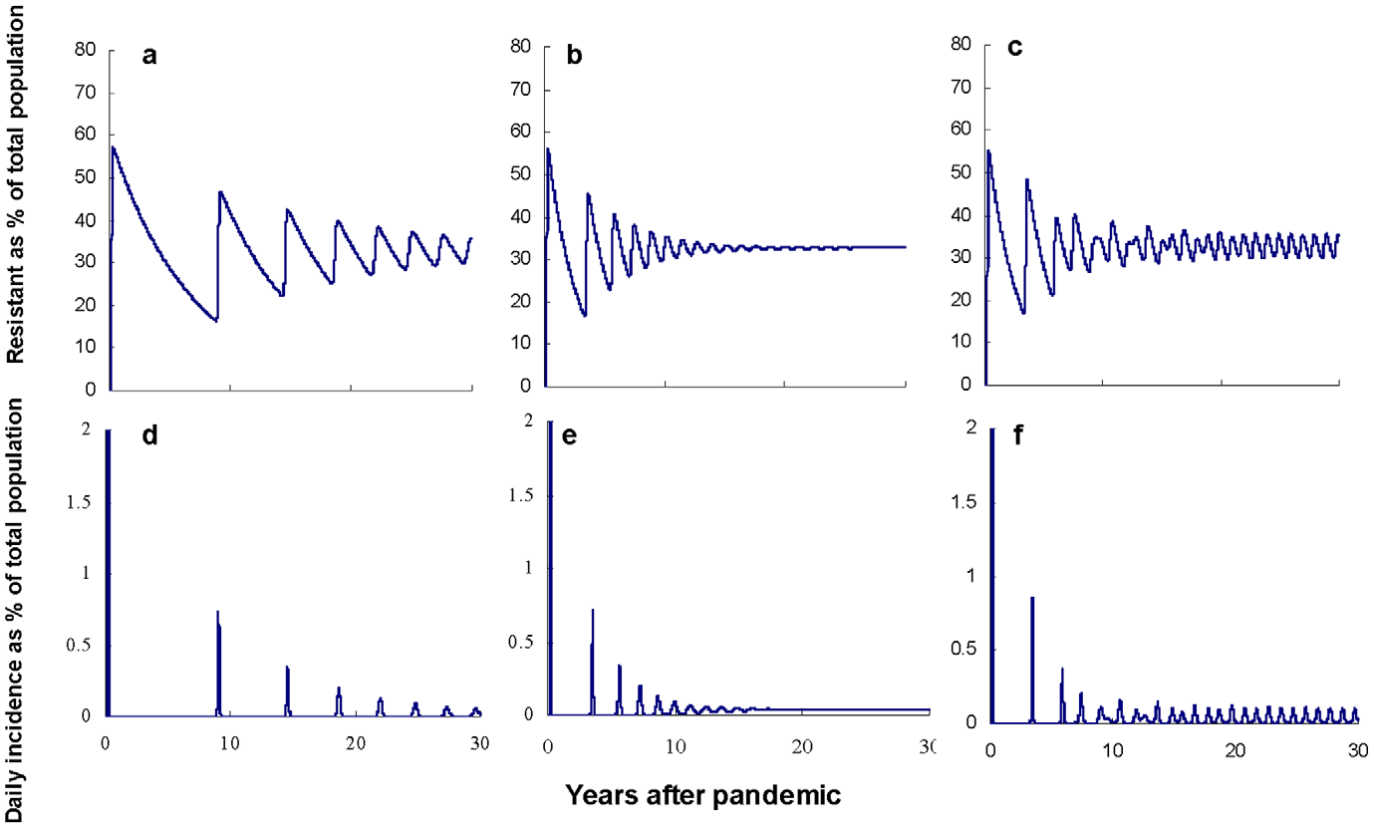
The impact of average immunity duration and the amplitude of seasonal forcing on the dynamics of pandemic and non-pandemic influenza. **a–c** show the proportion of the population that was resistant. **d–e** show the daily incidence as a proportion of the population (with the y-axis restricted so that the initial peak of incidence in the pandemic is not shown). **a** and **d** show the unforced dynamics (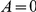) of the system with an average duration of immunity of 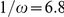 years (the average time between two cluster emergence events since the last pandemic in 1968, with clusters defined by [25]. The frequency of oscillations is still less than annual cycles 30 years after a pandemic. **b** and **e** show the unforced dynamics of the system with an average duration of immunity of 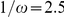 years. The frequency of oscillations increases to annual cycles within 10 years, but the lack of forcing results in flat non-seasonal incidence shortly afterwards. Note that the reduction of 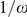 from 6.8 years to 2.5 years (or 2.5×364 days) has no substantial effect on the average proportion of the population that was immune (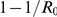) because the average duration of immunity is still much larger than the average duration of infectiousness (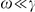) [10]. **c** and **f** show the baseline dynamics of our system with moderate seasonal forcing 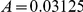 and an average duration of immunity at 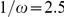 years. Seasonal forcing is strong enough to ensure the existence of a genuine off-season during which there are very few infections, but not strong enough to force the system into irregular non-annual cycles (such as those recently described for measles in Niger [30]. The combination of the degree of forcing and the natural frequency of the system permits low proportions of individuals resistant to influenza during immediately before the start of the season.

Some parameter values such as the average duration of the latent period 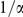 and the average duration of the infectious period 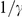 (Table 1) can be assumed with reasonable confidence from empirical studies. The value 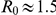 is consistent with a number of analyses of the 2009 pandemic ([2]; [21]). We adopted 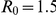 for our baseline and 1.8 as one of our sensitivity analyses. To capture the behaviour of severe pandemic as in Scandinavian cities in 1918 pandemic influenza [22], we examined other plausible value such as 5.4. However, there is still considerable uncertainty over the most appropriate values for some parameters in Model A: the average duration of effective immunity 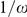 and the amplitude of seasonal forcing 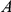. Therefore, we used solutions to Model A (Figure 1) to choose values for these parameters so that the resulting dynamics exhibit three characteristics typical influenza A epidemiology in temperate and subtropical climates. First, the fundamental frequency of the system falls close to annual cycles [12] within a short period after a pandemic. Second, the amplitude of seasonal forcing sufficiently strong that there is a genuine off-season with very little transmission. Third, the amplitude of seasonal forcing is weak enough that the system settles down into regular annual cycles: if seasonal forcing is too strong, there are frequently years with no infections. Although it would be desirable to estimate these unknown parameters using unbiased type-specific laboratory confirmed incidence data, such data are not currently available. Our exercise of parameterisation calibration was similar a recent exploratory analysis of influenza seasonality [23]. Also, our estimate of 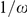 was also consistent with the estimate of the appearance of antigentic distinct clusters of other studies, ranged from 2–8 years[24], [25].

**Table 1:** Assumed values for transmission models.

Parameter		Value(s)	Notes
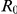	Basic reproductive number	1.5	Midpoint of estimates from [2], consistent with intervals from [19] and [18]
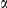	Reciprocal of average duration of latent period (days^−1^)	1/1.4	Analysis of a 2009 influenza A (H1N1) pandemic at a New York City school [27]
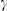	Reciprocal of average duration of infectiousness (days^−1^)	1/1.2	A generation time of 2.6 days [18] [28] and a latent period of 1.4 days implies an infection duration of 1.2 days for this SEIRS-type model [29]
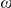	Reciprocal of average duration of immunity (days^−1^)	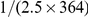	See Figure 1 and main text.
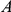	Amplitude of seasonal forcing	0.03125	See Figure 1 and main text.

## Results

We used household Model B to investigate the relationship between the relative infectivity within households 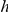, the proportion of infections that occurred within households during pandemic years 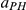, and the proportion of infections that occurred within households during non-pandemic years 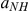 (Table 2). The first equilibrium non-pandemic year which is the first year to produce regular annual cycle of infection attack rate, by inspection, was year 15 in our baseline solution (see Figure 1). As 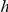 increased from 0.4 to 1.0, so did the proportion of infections that occurred in households (during both pandemic and non-pandemic years). However, the magnitude of the increase was not excessive. For example, doubling 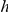 from 0.5 to 1.0 only increased the proportion of infections in households during pandemics from 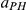 = 26.1% to 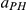 = 37.0%, and in non-pandemic years from 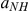 = 25.1% to 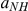 = 35.3%.

**Table 2:** Illustrative within-1 and between-household attack rates^2^ for pandemic and non-pandemic influenza under Ro = 1.5.

Infectivity ratio h	Household size	Pandemic attack rate		Non-pandemic attack rate	
		Total (% of population)	Within-house (% of Total)	Total (% of population)	Within-house (% of Total)
0.4	All	57.9	22.8	12.8	22.1
0.5		57.9	26.1	12.7	25.1
0.6		57.9	28.9	12.6	27.7
0.7	1	44.7	0	9.6	0
	2	50.0	25.5	11.8	24.0
	3	58.1	31.5	12.5	30.0
	4	59.8	34.5	12.9	33.1
	5	60.8	36.3	13.1	35.1
	All	57.9	31.3	12.5	30.0
0.8	All	57.9	33.4	12.4	32.0
0.9		57.9	35.3	12.3	33.7
1.0		57.9	37.0	12.2	35.3

1 The attack rate is defined as the number of infections divided by the total number of individuals in households of that size.

2 The within-household transmission is expressed as a percentage of the total number of infections.

**Figure 2:**
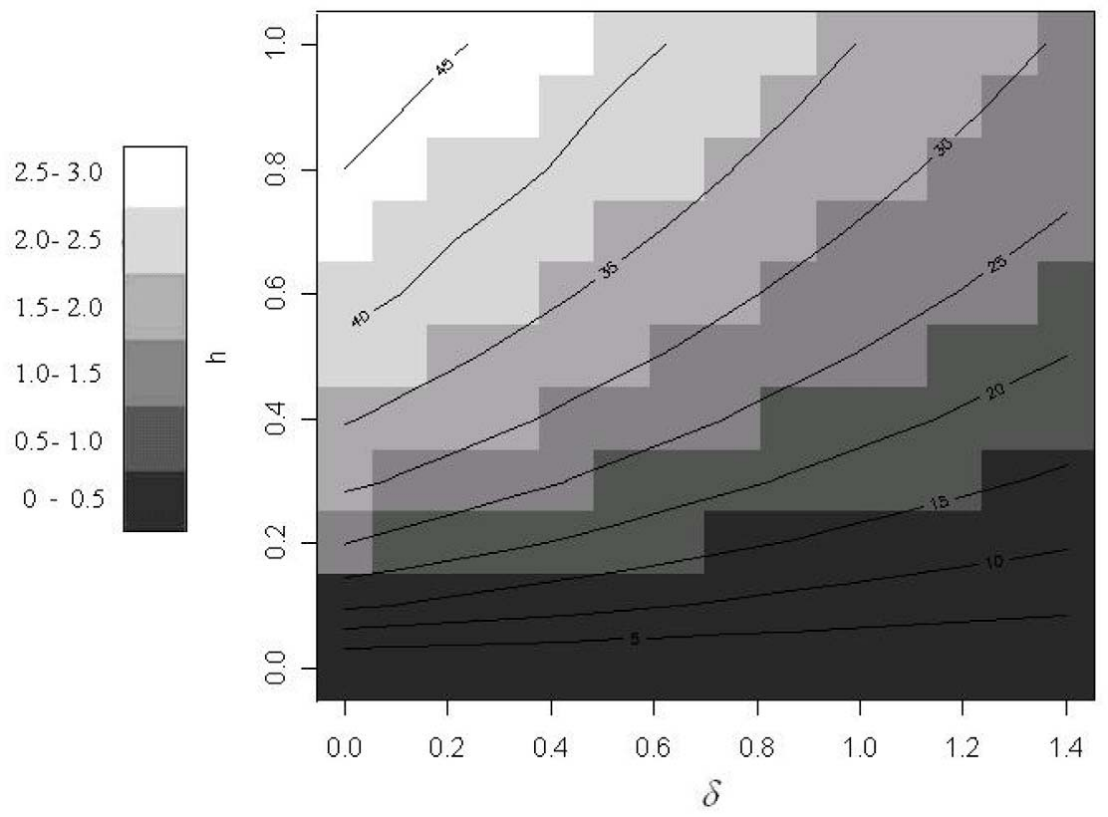
The within-household transmission percentages in non-pandemic years and the corresponding percentage difference from that in pandemic years. The contour lines show the percentages of within-household transmission in non-pandemic years, and the heat chart shows the differences between percentages of within-household transmission in non-pandemic years and pandemic years under different combinations of 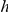 and 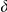. For example, if 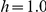 and 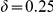, then the percentage of within-household transmission in a non-pandemic year would be 45% and the difference between the percentage of within household transmission in non-pandemic years and pandemic years would be approximately 2.5 to 3.0%.

We tested the sensitivity of our key findings to the nature of the force of infection within households not being genuinely pseudo-mass action (i.e. 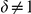, Figure 2). Small values of 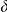 generated more infections within the household. Within the confidence bound for current estimates of 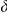, [0.46,1.21] [8], a large range of values of 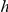 is consistent with a ∼30% annual attack rate in equilibrium years. In addition, the discrepancy between the proportion of infections that occur within the home during a pandemic year compared with an equilibrium year increases as the value of 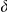 decreases.

**Figure 3:**
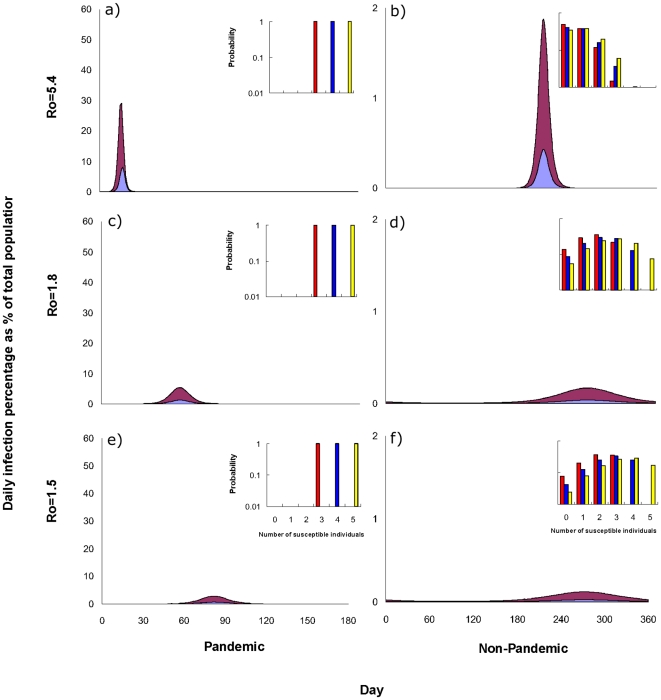
Proportion of within household and community infections in pandemic and non-pandemic years and the distribution of the number of susceptible individuals in non-pandemic year. Proportion of within-household (blue) and community (red) infections in the first six months of pandemic years (a, c and e) and non-pandemic years (b, d and f) and the distribution of the number of susceptible individuals from households of 3 (red) , 4 (blue) and 5 (yellow) at the start of non-pandemic years for different values of Ro: (a, b) 5.4 , (c, d) 1.8 and (e, f) 1.5.

Increasing the basic reproductive number R_0_ did alter the amplitude of the difference between pandemic and non-pandemic years. For size 5 households with R_0_ = 1.5, ∼47% had more than 3 susceptible individuals in non-pandemic years. With Ro values of 1.8 and 5.4 (Figure 3d and 3f), 28% and 1% of households, respectively, had more than 3 susceptible individuals. This implies that there would be less immunity in typical households of infected individuals with low R_0_ and more immunity in those with high R_0_ during non-pandemic years. This balancing results in a similar proportion of household infection across different R_0_ in non-pandemic years given similar proportions of household infection in pandemic years.

To reduce the sensitivity of our results to specific parameter choices, we also calculated some additional supporting model solutions (Tables 3 and 4). A sensitivity analysis on 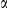 and 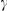 was performed by doubling and halving the values we used (1/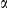 = 1.4 and 1/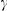 = 1.2) in Table 1. With 30% within-household attack rate in non-pandemic year, the percentage point differences in within house attack rate in non-pandemic years and non-pandemic years were very similar across different household sizes in Table 3. Further, different combinations of the seasonal forcing *A* and reciprocal of average duration of immunity 1/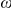 could produce similar within house attack rates in non- pandemic years. We incorporated three different levels of seasonal forcing on our dynamic transmission model. Thus, 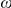 was estimated such that the regular annual cycle of infection was observed. With 30% within-household attack rate in non-pandemic year, the percentage point differences in within house attack rate are not substantial across different household sizes in three different scenarios (Table 4).

**Table 3:** Sensitivity analysis on duration of latent period and duration of infectiousness.

1/α	Household size	Non-pandemic (% of Total)			% point difference in within-house attack rate between pandemic and non-pandemic years (Pandemic attack rate - Non-pandemic attack rate)		
		1/γ					
		0.6	1.2	2.4	0.6	1.2	2.4
	1	0.0	0.0	0.0	0.00	0.00	0.00
	2	24.0	24.0	24.0	1.44	1.40	1.35
0.7	3	30.0	30.0	30.0	1.43	1.40	1.35
	4	33.1	33.1	33.1	1.35	1.31	1.27
	5	35.1	35.1	35.1	1.26	1.22	1.18
	All	30.0	30.0	30.0	1.31	1.27	1.21
	1	0.0	0.0	0.0	0.00	0.00	0.00
	2	24.0	24.0	24.0	1.50	1.45	1.49
1.4	3	30.0	30.0	30.1	1.48	1.44	1.47
	4	33.1	33.1	33.1	1.39	1.35	1.37
	5	35.1	35.1	35.1	1.29	1.25	1.26
	All	30.0	30.0	30.0	1.34	1.30	1.31
	1	0.0	0.0	0.0	0.00	0.00	0.00
	2	24.0	24.0	24.1	1.55	1.37	1.38
2.8	3	30.0	30.0	30.1	1.53	1.37	1.37
	4	33.1	33.1	33.2	1.42	1.28	1.28
	5	35.1	35.1	35.1	1.31	1.18	1.17
	All	30.0	30.0	30.0	1.37	1.22	1.21

**Table 4:** Sensitivity analysis on seasonal forcing.

Seasonal forcing A	Household size	Non-pandemic (% of Total)	% point difference of within-house attack rate in pandemic and non-pandemic years (i.e Pandemic attack rate - Non-pandemic attack rate)
0.015625	1	0	0
(1/ω = 3.4 years)	2	24.0	1.48
	3	30.0	1.47
	4	33.1	1.37
	5	35.1	1.27
	All	30.0	1.32
0.03125	1	0	0
(1/ω = 2.5 years)	2	24.0	1.50
	3	30.0	1.50
	4	33.1	1.40
	5	35.1	1.20
	All	30.0	1.30
0.0625	1	0	0
(1/ω = 1.4 years)	2	24.1	1.32
	3	30.1	1.31
	4	33.2	1.22
	5	35.2	1.13
	All	30.0	1.28

As a final observation, we note that differences in the distribution of susceptible individuals within households during non-pandemic years compared with pandemic years has important implications for the design of clinical trials of household-based interventions against influenza, especially those that recruit households via already infected members. In a population in which the transmission dynamics were well described by the scenario 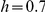 and 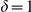, on average, the households of already infected individuals recruited after seeking health care would have few additional susceptible members. For example, using the steady-state solution of Model B with the abovementioned parameter values, we would expect only ∼57.5% of households of size 3 to have more than one susceptible individual (Figure 3).

## Discussion

We defined a plausible transmission scenario for an influenza pandemic and subsequent non-pandemic equilibrium seasons using a non-household mathematical transmission model (Model A). We then extended this to a model in which all household types were explicitly enumerated (Model B) and found that the proportion of infections which occurred in households during pandemics was similar to the proportion that occurred during non-pandemic years. Although this difference was somewhat sensitive to the nature of within-household transmission (pseudo-mass-action verses mass-action), the magnitude of the difference was not substantial for small values of R_0_ (say 1.4, 1.8). The sensitivity analysis on R_0_ (say R_0_ = 5.4) shows that higher R_0_ in pandemic year would show the difference of within household attack rate in pandemic and non-pandemic years.

The population-level efficacies of pandemic household-based mitigation strategies are sensitive to the proportion of infections that occur within households. Mitigation strategies are more effective when higher proportions of infections occur. It is thus reassuring that increased community force of infection did not generate a much lower proportion of infection within households during pandemics in our model, otherwise, the likely efficacy of household-based interventions in controlling a pandemic would be much reduced.

The household distribution of Hong Kong, as used in this study, is similar to those of many other developed nations [26]. However, less well developed populations and urban sub-populations in other countries have larger average household sizes. The study of the impact of household size distributions on infectious disease dynamics merits further theoretical and empirical study. Also, we chose not to include vaccination in this study. As an overall percentage, there are very few populations for which vaccination is a significant factor in the transmission of influenza. This was certainly the case during earlier empirical studies on which our parameters are based. If routine vaccination is extended to a large proportion of the population (or of school-age children) then future similar theoretical studies to that presented here should incorporate vaccination.

To simplify the model and increase its efficiency in solving hundreds of master equations, the asymptomatic class is excluded by assuming that the force of infections attributed by asymptomatic and symptomatic individuals are equal. The inclusion of asymptomatic individuals would improve the main results, but defining the hazard rate between these two groups to match the attack rates in pandemic years and inter-pandemic years would increase the uncertainties on extra parameter values.

The interpretation of our results is somewhat limited by our use of a deterministic framework for the transmission model. Our representation of an individual's loss of immunity is very simplistic – an exponential waiting time distribution from which every individual's loss of immunity is derived. We did not explicitly include births (which were “rolled” into the replenishment of susceptible individuals) and did not include step changes in the nature of the pathogen over time, which is sometimes the case for influenza. Thus, although our results are qualitatively robust, further studies are required to make more informed estimates of the scale of the effects we identified. For example, an individual-based stochastic version of these models and deterministic versions with step changes in the nature of the antigenic drift are worthy of investigation.

We have assumed that the basic reproductive number for influenza is the same in pandemic years as during non-pandemic years because we have no evidence to suggest the contrary. However, this equivalence may not be the case, especially during moderate or severe pandemics. To estimate the reproductive number for pandemic influenza, recent studies have re-examined time series of excess mortality from various cities in the United States and the United Kingdom during the 1918–1919 season [18], [19]. However, these populations may have been aware of the imminent arrival of a pandemic and may have already changed their behaviour to reduce average levels of transmission. The low estimates of R_0_ in these studies may not accurately reflect the mixing behaviour of populations during non-pandemic transmission periods. One might define a non-pandemic basic reproductive number for influenza as the average number of secondary cases generated by a typically infectious individual in an otherwise susceptible population in which people have not changed their mixing patterns in anticipation of a pandemic. We note that if the non-pandemic basic reproductive number is higher than that of a pandemic, it would substantially affect the non-pandemic attack rate (which is determined largely by the average duration of immunity).

Although the low constant R_0_ is assumed to be the case during past pandemics and non-pandemic years as mentioned above, we cannot rule out the possibility of the transmissibility of influenza with high constant R_0_ in the future. To have the household infection pattern for this scenario, a higher R_0_ of 5.4, for instance, could be incorporated into our model. Given 30% of household infection in non-pandemic years, the estimated *h* would be approximately 1.6. Based on this estimated *h*, quite different infection patterns would be observed in pandemic and non-pandemic years. A relatively small proportion of infections would be attributable to households in non-pandemic years than in pandemic years. For size 3 households, 37.9% and 28.5% would experience household infections in pandemic and non-pandemic years, respectively. This result implies that the current mitigation strategies recommended for households such as hand hygiene and mask wearing may not be effective enough to halt household transmission during pandemics in cases of high influenza transmission. Even for lower values of R_0_ such hygiene measures are unlikely to halt transmission too although to some extent, these measures will give a reduction in transmission.

It is unfortunate that we needed to rely on the broad properties of influenza transmission in non-pandemic years to characterize our baseline transmission scenario. This was necessary due to the lack of context-specific and unbiased laboratory confirmed incidence data for influenza in any population: we did not consider anonymous convenience samples from hospital surveillance networks and incidence estimated from excess seasonal mortality in all ages to be sufficiently accurate. Given the vast resources currently being consumed investigating vaccine and anti-viral candidates, we suggest that it is now appropriate to instigate multi-centre and multi-year population-based serological surveillance of influenza incidence. Such a program would be relatively inexpensive and could be used to rapidly address key shortfalls in our understanding of influenza epidemiology. If such accurate representative data were to become available, there would be great merit in extending the simple conceptual framework proposed here to include discrete changes in the serology of circulating strains and age specific transmission. It is likely, although not certain, that these refinements would need to be made within an individual-based stochastic version of our model. Systematically accurate incidence data and more refined transmission models could be used to make accurate population-specific estimates of influenza incidence conditional on the antigenic characteristic of the expected strain and of recent strains.
